# Sleep disturbances are associated with greater healthcare utilization in children with autism spectrum disorder

**DOI:** 10.1186/s11689-024-09550-z

**Published:** 2024-06-07

**Authors:** Shirley Solomon, Leena Elbedour, Gal Meiri, Analya Michaelovski, Yair Sadaka, Michal Ilan, Michal Faroy, Ilan Dinstein, Idan Menashe

**Affiliations:** 1 Azrieli National Centre for Autism and Neurodevelopment Research, Ben-Gurion University of the Negev, Beer-Sheva, Israel; 2 Department of Epidemiology, Biostatistics, and Community Health Sciences, Ben-Gurion University of the Negev, P.O. Box 653, Beer-Sheva, 84105 Israel; 3 Preschool Psychiatric Unit, Soroka University Medical Center, Beer-Sheva, Israel; 4 Child Development Center, Soroka University Medical Center, Beer-Sheva, Israel; 5 Child Development Center, Ministry of Health, Beer-Sheva, Israel; 6 Psychology Department, Ben-Gurion University of the Negev, Beer-Sheva, Israel; 7 Cognition and Brain Sciences Department, Ben-Gurion University of the Negev, Beer-Sheva, Israel; 8 Zlotowski Center for Neurosciences, Ben-Gurion University of the Negev, Beer-Sheva, Israel

**Keywords:** Autism spectrum disorder (ASD), Autism, Sleep, Sleep disturbances, Healthcare utilization, Co-occurring conditions, Medication use, Melatonin

## Abstract

**Background:**

Sleep disturbances are frequently reported in children with autism spectrum disorder (ASD) and are associated with the severity of co-occurring symptoms. This study’s aim was to examine the extent of healthcare utilization and clinical outcomes associated with sleep disturbances in children with ASD.

**Study design:**

A retrospective, cross-sectional study of 541 children with ASD from the Azrieli National Center for Autism and Neurodevelopment Research (ANCAN) whose parents completed the Children’s Sleep Habits Questionnaire (CSHQ). Children with a total CSHQ score ≥ 48 were defined as having sleep disturbances. Sociodemographic characteristics, ASD diagnostic measures, chronic co-occurring conditions, medication usage, hospitalizations, visits to the emergency room (ER), and visits to specialists were compared in ASD children with and without sleep disturbances. Multivariate logistic regression models were then used to assess the independent association of sleep disturbances with clinical characteristics and healthcare utilization.

**Results:**

Of the 541 children with ASD, 257 (47.5%) had sleep disturbances. Children with sleep disturbances exhibited higher rates of multiple (≥ 3) co-occurring conditions (19.1% vs. 12.7%; *p* = 0.0414) and prescribed medications (45.5% vs. 32.7%; *p* = 0.0031) than other children. Finally, ASD children with sleep disturbances were 1.72 and 2.71 times more likely to visit the ER and be hospitalized than their counterparts (aOR = 1.72; 99%CI = 1.01–2.95; and aOR = 2.71; 99%CI = 1.10–6.67, respectively).

**Conclusions:**

Our findings suggest that sleep disturbances are associated with greater healthcare utilization among children with ASD. Further studies could examine whether treating sleep disturbances in children with ASD yields additional clinical benefits beyond improvements in sleep.

**Supplementary Information:**

The online version contains supplementary material available at 10.1186/s11689-024-09550-z.

## Background

Adequate sleep is essential for normal brain development in children [1] and reduces the risk of mental health disorders [2], hypertension [3], obesity [3], and type-2 diabetes [4] in childhood and adolescence. In contrast, difficulties in initiating or maintaining sleep over a sustained period are associated with higher usage of medications, more frequent physician visits, and twice as many hospitalizations in the general population [5], as well as an increased risk of having at least one psychiatric or medical comorbidity [6].

Sleep disturbances, identified using parent reports, are reported in 40–80% of children with autism spectrum disorder (ASD) [7–9] in contrast to approximately 25% in typically developing children [10]. The Children’s Sleep Habits Questionnaire (CSHQ) [12] is a parent questionnaire that has been used widely to identify children with clinically meaningful sleep disturbances in typically developing and ASD children [13, 14]. The types of sleep disturbances reported in children with ASD include, but are not limited to, prolonged sleep onset, restless sleep, frequent awakenings, and a reduction in total sleep time [11, 12]. Higher rates of sleep disturbances in children with ASD relative to controls have been identified as early as 30 months of age and continue into adolescence [16].

Sleep disturbances have been associated with the severity of additional behavioral symptoms in children with ASD. For example, several studies reported that children with ASD and sleep disturbances also displayed higher sensory sensitivities [13–15] and more challenging behaviors, including irritability, hyperactivity, inattention, and hostility [16, 17]. Shorter sleep duration has also been associated with higher social communication difficulties and increased restricted and repetitive behaviors (RRBs) [18]. This has led to the suggestion that sleep disturbances may exacerbate the severity of core and secondary ASD symptoms [24, 25]. Thus, behavioral and pharmacological treatments for sleep disturbances in ASD may alleviate multiple symptoms [19]. Indeed, one recent study has reported that treatment with prolonged release of melatonin improved total sleep time and reduced hyperactivity in children with ASD [20].

Children with ASD are known to exhibit multiple co-occurring conditions and use healthcare services more frequently than controls. Specifically, it has been reported that children with ASD have a higher prevalence of gastrointestinal issues [21], seizures [22], epilepsy [23], and psychiatric conditions [24]. Furthermore, children with ASD utilize primary care (pediatric visits), specialty care (psychiatric, neurology visits), acute care (emergency room [ER] visits), outpatient care, and hospitalizations more frequently than controls [25, 26]. This increased healthcare may be attributable to the behavioral and developmental needs of children with ASD [33]. However, it may also be due to higher prevalence of co-occurring conditions [27]. For example, one study indicated that 13% of ER visits among children with ASD were psychiatric-related, compared to only 2% among children without ASD [28].

Given the higher rates of co-occurring conditions and healthcare utilization reported in children with ASD as well as the higher rates of sleep disturbances in these children, we aimed to examine the association between sleep disturbances and healthcare utilization in children with ASD registered in the database of the Azrieli National Center for Autism and Neurodevelopment Research (ANCAN).

## Methods

### Participants

We conducted a retrospective, cross-sectional study of 541 children with ASD between the ages of 1 and 10 years who were registered at the ANCAN database [29, 30] between 2015 and 2021. Children were included in the current study if they were members of Clalit Health Services (CHS) and their parents had completed the Children’s Sleep Habits Questionnaire (CSHQ). CHS is the largest health maintenance organization (HMO) in Israel, which insures 70% of the population in the south of Israel. We focused solely on members of CHS because the children’s electronic patient records from this HMO were available to us through the Soroka University Medical Center’s (SUMC) medical database. The study was approved by the SUMC Helsinki committee.

### Evaluation of sleep disturbances

Sleep disturbances were evaluated using the CSHQ, a 33-item parent questionnaire that asks parents to rate the frequency of specific sleep disturbances (e.g., difficulty to fall asleep) within the last week. The CSHQ was shown to exhibit high sensitivity and specificity in identifying children with clinical sleep disorders [12]. The CSHQ yields scores in eight subscales relating to common sleep disturbances: bedtime resistance, sleep onset delay, sleep duration, anxiety around bedtime, parasomnias, night wakening, sleep-disordered breathing, and daytime sleepiness. All items are summed to create a final total score ranging between 33 and 99, with higher scores indicating greater severity. We used a conservative threshold for identifying children with sleep disturbances using a CSHQ total score of ≥ 48 as suggested before [31]. The CSHQ has been used in research studies to assess children aged 4 to 10 years but has also shown to be clinically useful for screening sleep problems in younger children [32].

### Diagnosis and evaluation of core and secondary ASD symptoms

ASD diagnosis for all study participants was determined by a child psychiatrist or a pediatric neurologist according to the DSM-5 criteria following behavioral and cognitive assessments, as described previously [30]. Core ASD symptom severity was evaluated in all children using the Autism Diagnostic Observation Schedule, 2nd edition (ADOS-2) calibrated severity score (CSS). The ADOS-CSS, computed from ADOS-2 raw scores, allows comparison of ADOS-2 total scores across ages and modules [33]. We also compared symptom severity using the DSM-5 levels of required support (“Requiring support,” “Requiring substantial support,” “Requiring very substantial support”) in social communication (category A) and restricted, repetitive behaviors (RRB; category B) domains [34]. In addition, cognitive assessment scores from either the Bayley Scales of Infant and Toddler Development [35] or the Wechsler Preschool and Primary Scale of Intelligence (WPPSI) [36] were available for most (*n* = 445, 82%) of the children included in the final study sample.

### Evaluation of health services utilization and medication use

Health records were obtained from the CHS electronic patient record system for all participating children, as described before [27]. Co-occurring chronic conditions were obtained from the Ofek database that houses all medical data for every patient insured by CHS. This database documents, among other things, all chronic diagnoses that were recorded by primary care and specialist physicians. We extracted all co-occurring chronic conditions, which were coded according to the International Classification of Diseases, Ninth Revision (ICD-9) format, and grouped them into broader disease categories (excluding complications of pregnancy, childbirth, and the puerperium [630–676] and conditions originating in the perinatal period [760–799]). Codes 780–799, which are grouped as “symptoms, signs, and ill-defined conditions” according to the ICD-9, consist of symptoms, abnormal laboratory results or investigative procedures. This group includes labels such as “undiagnosed cardiac murmurs” and “respiratory abnormality, unspecified” and can otherwise be designated as “unknown etiology” [37]. Records of medication usage were obtained and grouped based on primary clinical use (Supplementary Table S1). In addition, hospitalizations, visits to the ER, and visits to specialists during a time period corresponding to one year before and after completion of the CSHQ were also gathered from the electronic records.

### Statistical analyses

Standard univariate tests were used to examine differences in various demographic and clinical characteristics between ASD children with and without sleep disturbances. Co-occurring chronic conditions and medication classes with a prevalence of less than 1% were excluded from the analyses. Chi-square or Fisher-exact tests were used to assess for differences in categorical variables, Mann–Whitney U‐test for continuous variables, and linear-by-linear association tests for ordinal variables. Differences with a p-value of < 0.05 were considered statically significant. Finally, the independent association between sleep disturbances and co-occurring chronic conditions, medication use, and healthcare utilization was assessed via multiple logistic regression models, each sequentially adjusting for potential confounders as follows. The initial analysis included only the sleep disturbances status (crude model). Basic sociodemographic and clinical covariates (e.g., age, sex, ethnicity, and DSM-V B required level of support) were then added to the model, followed by the addition of the presence of co-occurring conditions and medication use. The adjusted odds ratios of these associations were reported with stringent 99% confidence intervals. The statistical analyses were performed using R studio, version 1.4.1717 (R Foundation for Statistical Computing version).

## Results

Of the 1,108 children with ASD in the ANCAN database as of August 2021, 541 children (48.9%) fulfilled the study inclusion criteria. Children included in the sample were 3.25 (± 1.33) years old, on average, 79.1% of the children were of Jewish ethnicity, and their male-to-female ratio was 4:1. This sample did not differ significantly from the entire sample in the ANCAN database in these characteristics or in their cognitive scores, and ADOS-2 calibrated severity scores (Table 1). However, children in the study sample required more support than children who were not included in the study sample, as estimated by the diagnosing physician according to the DSM-5 levels of required support. This difference may reflect a tendency of parents of children who require more support to complete the CSHQ questionnaire.

**Table 1:** Comparison of characteristics across children with ASD registered in the National Autism Database of Israel who met inclusion criteria for the current study versus those who did not

Variables	Study Sample *n* = 541	Non-Study Sample *n* = 565	*P*-Value^a^
**Age at diagnosis (years)** ^**b**^	3.25 (1.33)	3.30 (1.4)	0.7318
**Sex**			0.3831
Male	430 (79.5%)	460 (81.6%)	
Female	111 (20.5%)	104 (18.4%)	
**Ethnic Origin**			0.5128
Jewish	416 (79.1%)	406 (77.3%)	
Bedouin	101 (19.2%)	113 (21.5%)	
Other	9 (1.7%)	6 (1.1%)	
**Cognitive Scores** ^**b**^	75.77 (18.3)	75.36 (17.1)	0.7692
**ADOS-2 CSS**	6.79 (2.3)	6.81 (2.4)	0.8787
**DSM-V A**	(*n* = 497)	(*n* = 496)	
Requiring support	67 (13.5%)	106 (21.5%)	**< 0.0001**
Requiring substantial support	201 (40.4%)	236 (47.9%)	
Requiring very substantial support	229 (46.1%)	151 (30.6%)	
**DSM-V B**			**< 0.0001**
Requiring support	84 (16.9%)	136 (27.4%)	
Requiring substantial support	231 (46.6%)	260 (52.4%)	
Requiring very substantial support	181 (36.5%)	100 (20.2%)	

Abbreviations: Autism Spectrum Disorder (ASD), Autism Diagnostic Observation Schedule, 2nd edition (ADOS-2)

Values are numbers of participants with percentages in parentheses, unless specified otherwise

a, Mann-Whitney U test for continuous variables, and χ^2^ test for categorical variables

b, Data are means ± standard deviations

**Fig. 1:**
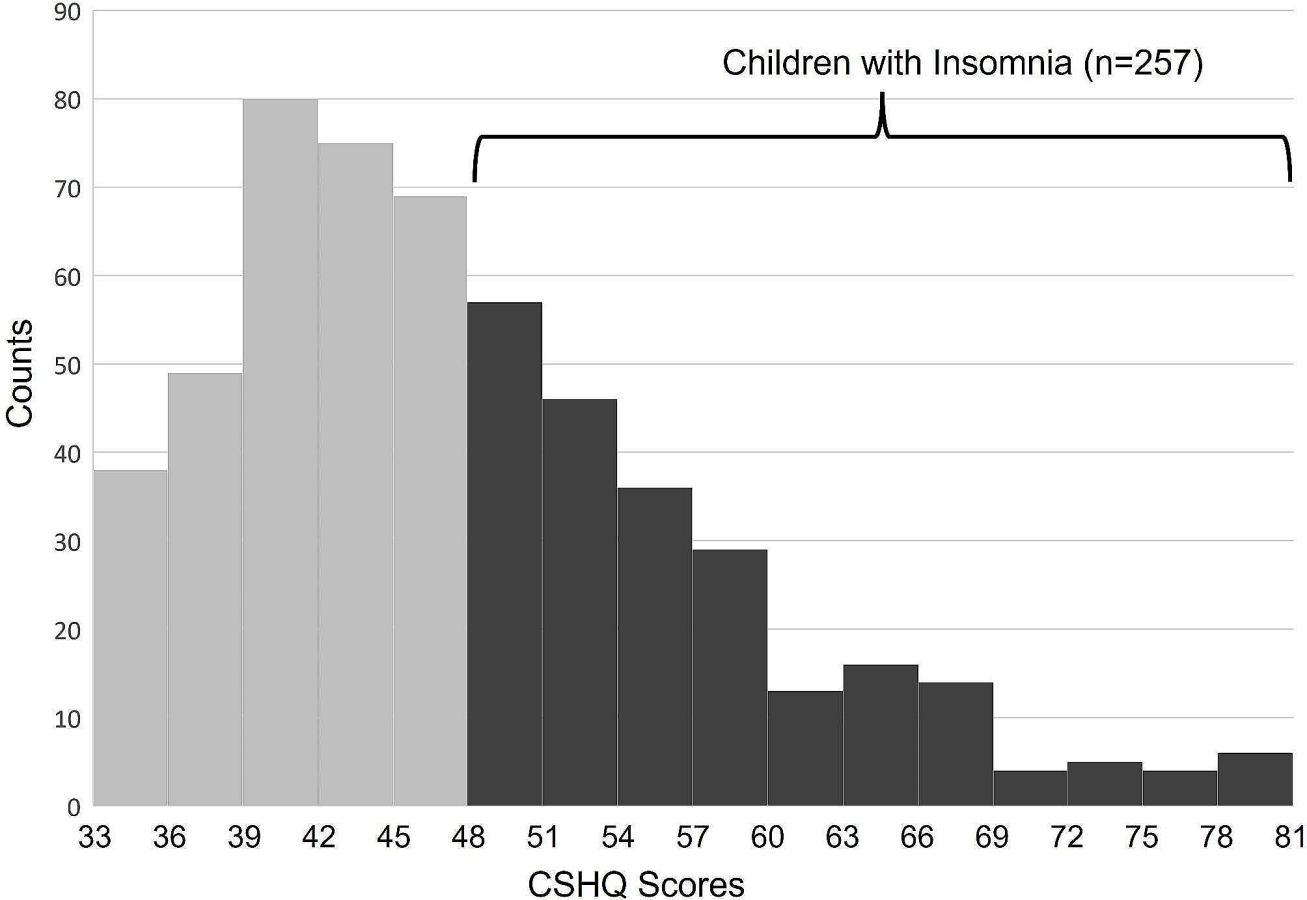
Distribution of CSHQ scores in the study sample. A histogram of the frequency of CSHQ scores (X-axis) of children with ASD in the study sample. Overall, 257 children had CSHQ scores ≥ 48 and were defined as having insomnia. Abbreviations: Children’s Sleep Habits Questionnaire (CSHQ)

Overall, participating children had a broad distribution of CSHQ scores ranging from 33 to 81, with 257 (47.5%) exhibiting CSHQ scores ≥ 48, which were indicative of sleep disturbances (Fig. 1). There were no significant differences in sex ratio, cognitive scores, or ADOS-2 calibrated severity scores across children with and without sleep disturbances (Table 2). However, more children of Bedouin origin were observed in the sleep disturbances group (24.6% vs. 14.2%; *p* = 0.01), and the mean age of the children in the sleep disturbances group was slightly higher (4.34 vs. 4.03 years; *p* = 0.039). In addition, children with sleep disturbances required more support according to the DSM-V criteria, with significant differences observed in the B criteria describing disturbances in RRB symptoms (*p* = 0.027).

**Table 2:** Comparison of characteristics across children with and without sleep difficulties

Sociodemographic Variables	No Sleep difficulties (CHSQ < 48) *n* = 284	Sleep difficulties (CSHQ ≥ 48) *n* = 257	*P*-Value^a^
**Age (years)** ^**b**^	4.03 (1.8)	4.34 (1.8)	**0.0394**
**Sex**			0.8763
Male	225 (79.2%)	205 (79.8%)	
Female	59 (20.8%)	52 (20.2%)	
**Ethnic Origin**			**0.0101**
Jewish	230 (83.9%)	186 (73.8%)	
Bedouin	39 (14.2%)	62 (24.6%)	
Other	5 (1.8%)	4 (1.6%)	
**Cognitive Scores** ^**b**^	76.46 (17.2)	74.92 (19.6)	0.5275
**ADOS-2 CSS**	6.67 (2.3)	6.94 (2.3)	0.1906
**DSM-V A**			
Mild	36 (14.0%)	31 (13.0%)	0.1955
Moderate	112 (43.4%)	89 (37.2%)	
Severe	110 (42.6%)	119 (49.8%)	
**DSM-V B**			**0.0278**
Mild	47 (18.3%)	37 (15.5%)	
Moderate	130 (50.6%)	101 (42.3%)	
Severe	80 (31.1%)	101 (42.3%)	

Abbreviations: Autism Spectrum Disorder (ASD), Autism Diagnostic Observation Schedule, 2nd edition (ADOS-2), Children’s Sleep Habits Questionnaire (CSHQ),

Values are numbers of participants with percentages in parentheses, unless specified otherwise

a, Mann-Whitney U test for continuous variables, and χ^2^ test/fisher’s exact test for categorical variables

b, Data are means ± standard deviations

### Co-occurring chronic conditions

Differences in *co-occurring chronic conditions* are depicted in Table 3. There was a small but non-significant difference in number of ASD children who had any co-occurring chronic condition (49.8% vs. 45.8%; *p* = 0.3947) between the study groups. However, more ASD children with sleep disturbances had three or more co-occurring conditions compared to children without sleep disturbances (19.1% vs. 12.7%; *p* = 0.0414). In addition, ASD children with sleep disturbances had significantly more co-occurring conditions in the symptoms, signs, and ill-defined conditions classification than ASD children without sleep disturbances (21.0% vs. 13.7%, *p* = 0.0334). There were no significant differences in other examined categories of co-occurring chronic conditions.

**Table 3:** Comparison of co-occurring chronic conditions between ASD children with and without sleep difficulties

		No Sleep difficulties (CHSQ < 48) *n* = 284	Sleep difficulties (CSHQ ≥ 48) *n* = 257	*P*-Value^*^
**Any Co-occurring Chronic Conditions**		130 (45.8%)	128 (49.8%)	0.3947
**Number of Co-occurring Chronic Conditions**	**1**	59 (20.8%)	50 (19.5%)	0.7024
	**2**	35 (12.3%)	29 (11.3%)	0.7084
	**≥ 3**	36 (12.7%)	49 (19.1%)	**0.0414**
Endocrine, Nutritional, Metabolic, and Immunity		14 (4.9%)	15 (5.8%)	0.7821
Blood and Blood-Forming Organs		16 (5.6%)	13 (5.1%)	0.9159
Mental Disorders		15 (5.3%)	22 (8.6%)	0.1808
Nervous System and Sense Organs		31 (10.9%)	35 (13.6%)	0.4078
Respiratory System		29 (10.2%)	33 (12.8%)	0.4102
Digestive System		5 (1.8%)	8 (3.1%)	0.4565
Skin and Subcutaneous Tissue		9 (3.2%)	16 (6.2%)	0.1373
Musculoskeletal System and Connective Tissue		4 (1.4%)	5 (1.9%)	0.7421
Congenital Anomalies		15 (5.3%)	22 (8.6%)	0.1808
Symptoms, Signs, And Ill-Defined Conditions		39 (13.7%)	54 (21.0%)	**0.0334**
Injury and Poisoning		24 (8.5%)	25 (9.7%)	0.7138
*Developmental Delay*		22 (7.7%)	21 (8.1%)	0.9815

Abbreviations: Autism Spectrum Disorder (ASD), Children’s Sleep Habits Questionnaire (CSHQ)

Values are numbers of participants with percentages in parentheses

^*^ χ^2^ test or Fisher exact tests were conducted to determine the association between chronic comorbidities and CSHQ score

### Medication use

The use of medication for the management of chronic conditions (as listed in Supplementary Table S1) for both groups is presented in Table 4. Children with sleep disturbances were more likely to be prescribed medications than those without (45.5% vs. 32.7%, *p* = 0.0031). This difference was partially due to medication prescriptions for sleep disturbances (e.g., Melatonin and Promethazine) that were almost twice as frequent in the sleep disturbances group (15.2% vs. 8.1%, *p* = 0.0145). Nevertheless, the number of prescribed medications for children with ASD and sleep disturbances was still significantly higher after excluding medications that treat sleep disturbances (37.0% vs. 28.2%, *p* = 0.0364), demonstrating their higher use of medications unrelated to sleep disturbances. Specifically, prescriptions for medications in the treatment of mental or mood conditions were significantly more frequent in the sleep disturbances group (9.7% vs. 4.9%, *p* = 0.0468).

**Table 4:** Comparison of medication usage between ASD children with and without sleep difficulties

Prescribed Medications^a^	No Sleep difficulties (CHSQ < 48) *n* = 284	Sleep difficulties (CSHQ ≥ 48) *n* = 257	*P*-Value
**Any Medications**	93 (32.7%)	117 (45.5%)	**0.0031** ^**b**^
**Medications for Sleep Difficulties**	23 (8.1%)	39 (15.2%)	**0.0145** ^**b**^
**Medications, Excluding those for Sleep Difficulties**	80 (28.2%)	95 (37.0%)	**0.0364** ^**b**^
**Asthma/Pulmonary Disorders**	30 (10.6%)	30 (11.7%)	0.7845^**b**^
**ADHD**	30 (10.6%)	26 (10.1%)	0.9769^**b**^
**Mental and Mood Conditions**	14 (4.9%)	25 (9.7%)	**0.0468** ^**b**^
**Nutrient Deficiencies**	14 (4.9%)	20 (7.8%)	0.2349^**b**^
**Food supplements**	9 (3.2%)	16 (6.2%)	0.1373^**b**^
**Seizure Disorders**	6 (2.1%)	8 (3.1%)	0.6451^**b**^

Abbreviations: Autism Spectrum Disorder (ASD), Children’s Sleep Habits Questionnaire (CSHQ), Attention Deficit Hyperactivity Disorder (ADHD)

Values are numbers of participants with percentages in parentheses

^a^ Medications were grouped based on primary clinical use (Supplementary Table S1)

^b^ Pearson Chi-Square test. ^c^ Fisher-Exact test

### Health services utilization

**Fig. 2:**
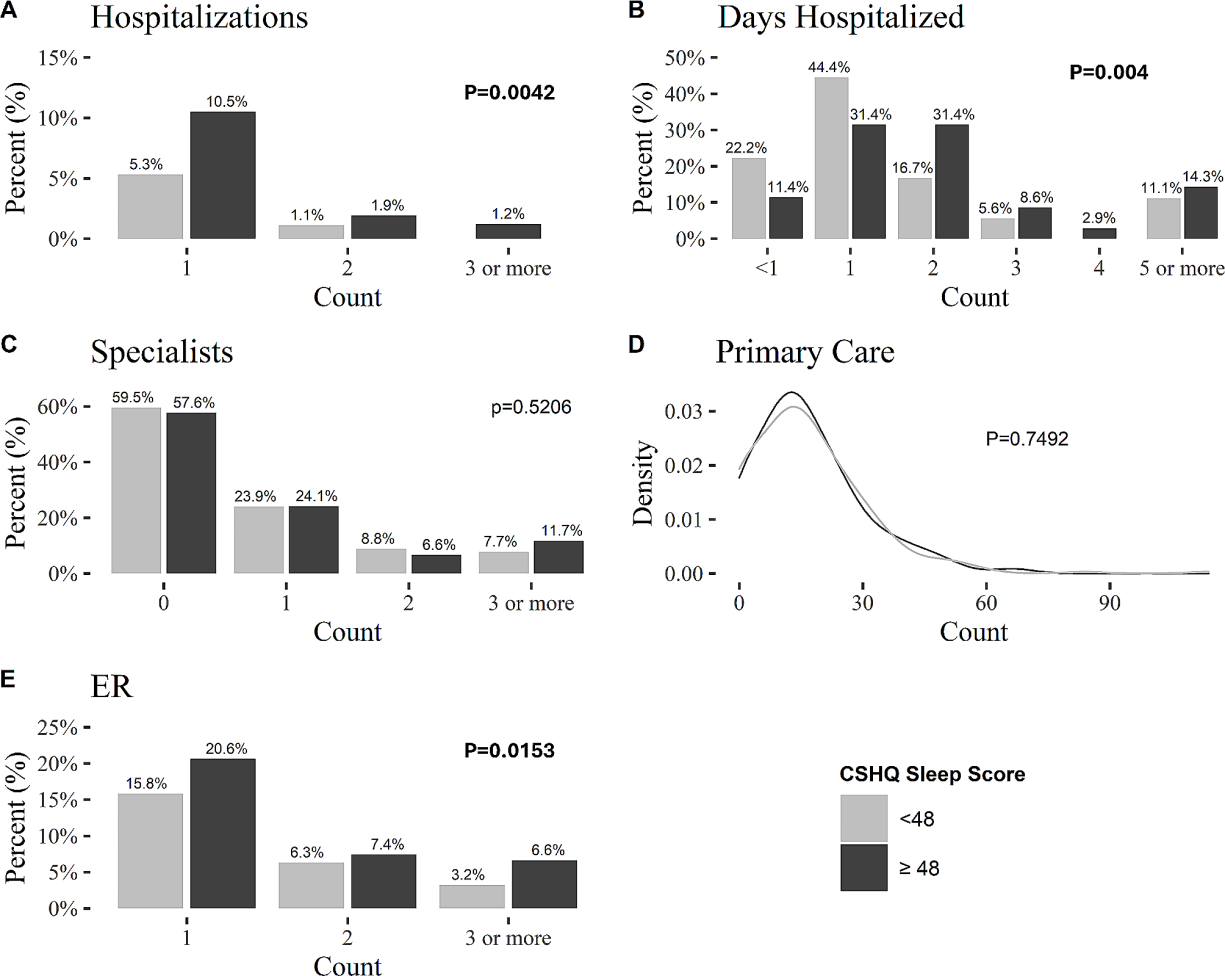
Percentage of health services utilization during a period of one year before and after completion of the CSHQ. **A.** Number of hospitalizations. **B.** Days hospitalized. **C.** Number of visits to a specialist. **D**. Number of visits to a primary care physician. **E**. Number of visits to the ER. Note that percentages presented in panels A, C, and E sum to 13.6%, 42.4%, and 34.6% of ASD children with insomnia and 6.4%, 58.5%, and 25.3% of children without insomnia who were hospitalized, visited specialists, and visited the ER, respectively. P-values from Mann-Whitney U tests are for the differences between children with and without insomnia. Abbreviations: Children’s Sleep Habits Questionnaire (CSHQ), ER (ER)

Children with ASD and sleep disturbances utilized more health services than children without sleep disturbances (Fig. 2), including 50% more visits to the ER (mean[SD] = 0.63[1.19] vs. 0.42[1.01]; *p* = 0.0153) and a 2.7 times higher rate of hospitalization (mean[SD] = 0.19[0.60] vs. 0.07[1.30]; *p* = 0.0042). Moreover, children with sleep disturbances were hospitalized for twice the number of days compared to children without sleep disturbances (mean[SD] = 0.32[1.08] vs. 0.16[1.06] days per child, respectively; *p* = 0.004). These findings suggest that the cost of ER visits and hospitalization is significantly higher for children with sleep disturbances. No significant differences were found in the total number of outpatient visits, including visits to primary care physicians (mean [SD] = 16.7 [14.7] vs. 16.9 [13.2] visits per child, respectively; *p* = 0.7492) and specialists (mean[SD] = 0.95[1.81] vs. 0.80[1.51] visits per child, respectively; *p* = 0.5206).

### Factors associated with sleep disturbances

Finally, we used multivariable logistic regression models to quantify the independent association of sleep disturbances with co-occurring chronic conditions, medication use, and healthcare utilization in our sample while controlling for potential confounders (Table 5). Sleep disturbances in children with ASD were associated with higher odds of having co-occurring chronic conditions and medication use. However, these associations were not statistically significant in the fully adjusted models (Table 5). Nevertheless, children with sleep disturbances were 1.72 more likely to visit the ER (≥ 1 visits during the study period) (aOR = 1.72; 99% CI = 1.01, 2.95), and 2.71 more likely to be hospitalized (≥ 1 hospitalizations during the study period) (aOR = 2.71; 99% CI = 1.10, 6.67), even after adjusting for sociodemographic characteristics, presence of co-occurring chronic conditions and medication use.

**Table 5:** Factors associated with sleep difficulties in children with ASD.

	Crude OR (99% CI)	Adjusted OR^a^ (99%CI)	Adjusted OR^b^ (99%CI)	Adjusted OR^c^ (99%CI)
**Co-occurring Chronic Conditions**	1.18 (0.75, 1.83)	1.04 (0.64, 1.69)		
Symptoms, Signs, And Ill-Defined Conditions	1.67 (0.92, 3.03)	1.70 (0.90, 3.22)		
Multiple condiitons (3 or more)	1.62 (0.88, 3.00)	1.58 (0.81, 3.08)		
**Medication Use**	**1.72** **(1.09, 2.71)**	1.47 (0.89, 2.43)	1.36 (0.90, 2.62)	
Medications for sleep difficulties (Melatonin and Promethazine)	2.03 (0.99, 4.16)	1.72 (0.80, 3.70)	1.72 (0.80, 3.70)	
Medications, excluding those for sleep difficulties	1.50 (0.93, 2.41)	1.28 (0.76, 2.15)	1.31 (0.74, 2.31)	
Mental and Mood Conditions	2.08 (0.85, 5.06)	1.62 (0.59, 4.47)	1.62 (0.58, 4.51)	
**Emergency Room Visits (Yes/No)**	1.56 (0.96, 2.54)	**1.75** **(1.03, 2.98)**	**1.76** **(1.03, 3.00)**	**1.72** **(1.01, 2.95)**
**Primary Care Visits (Yes/No)**	1.00 (0.98, 1.02)	1.00 (0.98, 1.02)	1.00 (0.98, 1.02)	1.00 (0.99, 1.02)
**Specialist Visits (Yes/No)**	1.08 (0.69, 1.70)	1.21 (0.74, 1.98)	1.21 (0.74, 1.98)	1.17 (0.71, 1.91)
**Hospitalizations (Yes/No)**	**2.33** **(1.06, 5.10)**	**2.82** **(1.15, 6.89)**	**2.83** **(1.15, 6.94)**	**2.71** **(1.10, 6.67)**

Abbreviations: Autism Spectrum Disorder (ASD), Children’s Sleep Habits Questionnaire (CSHQ), Odds Ratio (OR)

ORs were calculated using the logistic regression formula: *Ln (Odds(Yi)) = a + biXi*, where i = 1 for sleep disturbances and i = 0 for no sleep disturbances

^a^ Adjusted for sociodemographic variables (sex, ethnicity, age, and DSM-V category B)

^b^ Additionally adjusted for chronic comorbidities

^c^ Additionally adjusted for medication use

## Discussion

The results demonstrate that sleep disturbances are present in nearly 50% of children with ASD. These children have a higher number of co-occurring chronic conditions, use more medications, and are more likely to visit the ER and be hospitalized compared to ASD children without sleep disturbances.

These findings from children with ASD are in line with previous studies that have reported positive associations between sleep disturbances and a diverse range of co-occurring chronic conditions within the general adult population. These include associations between sleep disturbances and anxiety and depression [38], gastrointestinal disorders [39], psychopathy symptoms [40], and even a 45% increased risk of mortality from cardiovascular disease [41]. The mechanism underlying the associations between sleep disturbances and co-occurring chronic conditions remains unknown. It is possible that the symptoms associated with co-occurring conditions (e.g. anxiety or gastrointestinal disorders) cause sleep disturbances. Alternatively, sleep disturbances could increase the risk of developing or exacerbating co-occurring chronic conditions. A third option is that alterations in biological mechanisms such as circadian rhythm misalignment [42], low-grade inflammatory state [43], elevation of cortisol [44], and metabolic or endocrine changes [45] could contribute to both sleep disturbances and co-occurring chronic conditions. Some of these mechanisms were also suggested to be involved in ASD etiology [46, 47], thus highlighting potential mechanistic links between ASD and sleep disturbances.

The association of sleep disturbances with greater use of health services in this study is also consistent with previous reports of increased utilization of health services with sleep disturbances in the general adult population, including more frequent ER visits and hospitalizations, but no significant increases in the number of physician visits [5, 48, 49]. Moreover, in our study, sleep disturbances were independently associated with healthcare utilization in ASD children even after controlling for medication use and chronic co-occurring chronic conditions. In a previous study we reported that children with ASD are referred to the ER and admitted to the hospital more frequently than children without ASD [27]. It is possible that the higher prevalence of sleep disturbances in children with ASD compared to controls contributed to this observation. Furthermore, the association between sleep disturbances and healthcare utilization was also demonstrated in other studies with an adult population, where the association between sleep disturbances and increased hospitalizations and ER visits remained significant even after accounting for medical or mental health conditions [50, 51]. A possible explanation for the association between sleep disturbances and hospitalizations and ER visits, but not with outpatient or specialist visits, may be due to the nature of ER visits. Sleep disturbances have been shown to increase the amount of challenging behaviors including hyperactivity, irritability and hostility [16, 17], potentially increasing the risk for injuries that may require a visit to the ER. Unfortunately, we could not test this hypothesis due to the lack of information regarding the reason for ER admission in the medical records used in this study.

Finally, ASD children with sleep disturbances were more likely to consume more medications for sleep as well as other medications than ASD children without sleep disturbances. These findings are consistent with similar differences between good and bad sleepers in the general population [53, 54]. Notably, in the current study, the association between medication use and sleep disturbances was statistically significant only in the unadjusted regression model, and this association, although it remained positive, lost its statistical significance in the fully adjusted model. This finding is expected given that ASD children with sleep disturbances display more challenging behaviors [16, 17], and antipsychotics, including risperidone and aripiprazole (the two most common medications prescribed in this study sample under the mental and mood conditions classification), are prescribed to reduce challenging behaviors, particularly irritability and aggression in children with ASD [55, 56].

The results reported in this study should be interpreted in the context of the following limitations. First, information on the child’s sleep behavior was gathered via parental reports using the CSHQ. This method may be biased as it relies on parents’ perception and their subjective ability to recall their child’s sleep disturbances accurately. Alternative methods such as daily sleep diaries or direct measures such as actigraphy and polysomnography may offer less biased estimates of sleep disturbances; however, these measures are often difficult to acquire from children with ASD due to sensory sensitivities and lack of cooperation [58]. Second, this study used a retrospective, cross-sectional design. Thus, it was impossible to determine the causality or directionality of the association between the emergence of sleep disturbances and the utilization of health services and/or clinical outcomes. It is unknown whether sleep disturbances directly increase the risk of these factors or if adverse health outcomes produce sleep disturbances, as a temporal relationship was not established. It is also possible that sleep disturbances and health outcomes are caused by a shared underlying biological mechanisms. Third, data was obtained solely from the electronic records, which included only clinical data, and no information regarding use of over-the-counter medications, including melatonin. Furthermore, no information was provided regarding the nature of ER visits. Fourth, despite a large sample size, the rarity of some co-occurring chronic conditions and prescribed medication classes limited our ability to find a significant association between sleep disturbances and these variables. Indeed, a post-hoc power analysis indicated that the observed differences between the study groups in the rates of each of the co-occurring chronic conditions and medication classes only had a 40% statistical power to declare these differences as statistically significant within our sample size. Finaly, data collection was derived from children living in southern Israel and enrolled in a single HMO, which may have limited the generalizability of the study findings to other populations.

## Conclusions

Our findings suggest that sleep disturbances are associated with greater healthcare utilization among children with ASD. Future studies should validate our findings and further investigate the underlying mechanisms of these associations. Furthermore, it would be interesting to examine whether treatments for sleep disturbances reduce the utilization of health services in a manner associated with the amelioration of sleep problems in children with ASD.

### Electronic supplementary material

Below is the link to the electronic supplementary material.

Supplementary Material 1

## Data Availability

The datasets generated and/or analyzed during the current study are not publicly available due to ethical reasons. The anonymized data could be obtained from the corresponding author on reasonable request.

## Abbreviations

ASD: Autism Spectrum Disorder
ANCAN: Azrieli National Center for Autism and Neurodevelopment Research
CSHQ: Children’s Sleep Habits Questionnaire
RRB: Repetitive Behavior
ER: Emergency Room
CHS: Clalit Health Services
HMO: Health Maintenance Organization
SUMC: Soroka University Medical Center’s
